# Enhanced anaerobic digestion of freezing and thawing pretreated cow manure with increasing solid content: kinetics and microbial community dynamics

**DOI:** 10.1038/s41598-024-76392-z

**Published:** 2024-10-26

**Authors:** Muhammad Abid, Jing Wu, Yan Yuanyuan, Zeeshan Ajmal, Tariq Mehmood, Syed Nabeel Husnain, Xu Zhou

**Affiliations:** 1 International Faculty of Applied Technology, Yibin University, Yibin, 644000 Sichuan China; 2 State Key Joint Laboratory of Environment Simulation and Pollution Control, School of Environment, Tsinghua University, Beijing, 100084 China; 3 Beijing Zhongchi Green Energy Environmental Technology Co., Ltd, Beijing, China; 4 School of Chemistry and Material Science, Zhejiang Normal University, Jinhua, China; 5 Department Sensors and Modeling, Potsdam de Leibniz Institute for Agricultural Engineering and Bioeconomy (ATB), Max-Eyth-Allee 100, 14469 Potsdam, Germany; 6 Department of Energy Systems Engineering, University of Agriculture Faisalabad, Faisalabad, Pakistan

**Keywords:** Pretreatment, High solid anaerobic digestion, Biomethane, Microbiology, Methanogenic pathways, Environmental sciences, Energy science and technology

## Abstract

High solid anaerobic digestion has proved the mainstream technology for the treatment of organic wastes. However, the high molecular weight and complex lignocellulosic structure of cow manure (CM) make it indigestible and inefficient, leading to limit the hydrolysis step of anaerobic digestion at high solid content. To mitigate this bottleneck, an improved cost-effective freezing and thawing pretreatment technique was proposed in this study. The freezing and thawing pretreatment of raw CM without any dilution was done for 20 days. The maximum cumulative methane yield (487 mL CH_4_ g^− 1^VS) was achieved at a total solid (TS) of 5% followed by TS of 10% and 15%, which was 13%, 20% and 21% higher than obtained from untreated CM, respectively. The kinetic results revealed that the biodegradable materials could be utilized at increasing TS with decreasing hydrolysis rate. The pretreatment significantly enhanced the methylotrophic methanogenic pathway during high solid anaerobic digestion, which was contrary to the general concept that the process is usually dominated by acetoclastic and hydrogenotrophic methanogens. This study is very important to understand the effect of solid content but also important to understand the effect of freezing and thawing pretreatment on process parameters and microbial community dynamics in high solid anaerobic digestion.

## Introduction

The tremendous increase in population and urbanization have increased the demand for food, water, shelter and energy. A large amount of organic waste is generated each day worldwide, putting great pressure on municipalities and polluting our land, water and air. It is estimated that the world’s annual manure production is between 15 and 20 million metric tons^1^. High solid anaerobic digestion (HSAD) has proved prevalent technology for the utilization of organic wastes however, the higher molecular weight and complex lignocellulosic nature of cow manure (CM) makes it a rate-limiting step during the HSAD process. The lignocellulosic substances are the largest proportion of CM hence, making it challenging during mono-digestion^2^ hereafter, causing long solid retention time (SRT), large reactor volume, toxic intermediates, poor methanogenesis and ultimately lowering biomethane yield.

The above impediments can be dealted by adopting the pretreatment of CM before HSAD process. For instance, the methane potential of thermochemically pretreated CM (10% of NaOH at 100 °C for 5 min) was enhanced by 23.6%^3^. When CM with 15.9% TS was subjected to mechanical pretreatment via shredding and mixing (5 min at 20 °C) and then finally blending (by adding water to get a water/dry matter ratio of 10), the methane production and rate were significantly increased (11% and18% respectively) between untreated and blended samples^4^. The methane yield of CM was improved by 127% by thermal pretreatment with 3% NaOH pretreated sample at 180 °C. The results showed that cellulose, hemicellulose and lignin were ideally reduced by 24.8%, 29.1% and 9.5% respectively during pretreatment and 76.5% of cellulose and 84.9% of hemicellulose were converted to methane during AD^5^. Although the physicochemical pretreatments can enhance biogas yield to some extent but these techniques usually rely on high energy inputs and high capital cost. To avoid or minimize these constrictions of physicochemical pretreatment, it is imperative to develop an efficient, environmentally friendly and economically viable pre-treatment approach.

Recently, psychrophilic low-temperature pretreatment techniques with simple operating procedures were applied to produce ethanol and glucose from the biomass. For instance, corn stover at 30% solid content when pretreated at -20 °C produced 261 mL g^− 1^VS biomethane Which was 41.08% higher than control^6^. The high cellulosic fractions dissolved in 7% NaOH at -12° C enhanced ethanol yield from 75.1 to 77.7%^7^. Zhang et al.^8^ studied the effects of freezing-thawing pretreatment on AD of wheat straw at TS 96.94% with different temperatures and time gradients (− 10 °C, − 20 °C, − 40 °C and − 80 °C and 12 h, 24 h, 48 h and 96 h. respectively), the results showed that pretreatment can shorten the lag phase time of AD, providing a 21.39% improvement under the optimal conditions of − 20 °C 96 h compared to the control. Moreover, freezing and thawing pretreatment technologies have been successfully employed for the production of glucose, ethanol and biomethane in recent years^9^.

Based on the above literature, it can be incurred that freezing and thawing pretreatment can considerably improve the solubility of lignocellulosic fibers, thus could increase the hydrolysis rate and biomethane yield. However, under natural conditions freezing and thawing pretreatment has not been optimized for fresh CM with increasing total solid (TS) during the HSAD process. Owing to the special physicochemical properties, CM is considered the most feasible substrate for full-scale AD plants^10^, however, the presence of the hardly degradable lignocellulosic fractions has limited hydrolysis step resulting in prolonged SRT and large reactor volume hence this issue is worth researching.

In addition, microorganisms play a very crucial role in the HSAD process. The physicochemical characteristics of feedstock can significantly influence the population, diversity, activity and structure of the microbial community in the AD system. Some pretreatment techniques can adversely affect the microbial community during the AD process. For example, thermochemical pretreatment approaches can cause inhibition for both archaeal and bacterial communities by releasing inhibitory substances like furfural, vanillin, 5-HMF and some other phenolic substances^11^. In literature, few studies have focused on the relationship between pretreatment and microbial community dynamics during AD of different substrates and there is a lack of information about the effect of freezing and thawing pretreatment on HSAD of CM in natural conditions.

Therefore, the current study aimed to investigate the effect of natural freezing and thawing pretreatment on fresh CM. This pretreatment technology is eco-friendly and economically viable because no chemical or catalyst is used during the pretreatment. It can be adopted in a wide range of countries with freezing and thawing weather conditions, especially in wet and partially moistened environments. These conditions enable biomass to get moisture and natural freezing and thawing phenomenon takes place in feedstock due to variations in atmospheric temperature. Hence, no additional energy is required as compared with other pretreatment technologies. In particular, this study first time, investigates the seasonal change in temperature and its ability to degrade lignocellulosic substances in undiluted CM for enhanced HSAD process with increasing solid content. Moreover, this study first time briefly explains the effect of pretreatment on microbial community dynamics during HSAD of CM and their metabolic pathways.

## Materials and methods

### Substrate and inoculum

The CM was collected in December 2020 from a dairy farm located in Shandong province of China. The plastic airtight buckets were used to carry inoculum from continuously operated mesophilic (35–40 °C) full-scale HSAD plant treating CM in Taian City of Shandong province Mainland China. The main constituents and characteristics of the raw CM (RCM) pretreated CM (PCM) and inoculum are summarized in Table 1.

**Table 1:** Characteristics of raw CM and inoculum during pretreatment study.

Parameters	Inoculum	RCM	PCM
pH	8.02 ± 0.7	7.7 ± 0.03	7.21 ± 0.05
TS*	118.49 ± 0.63	249.82 ± 0.27	198.16 ± 0.44
VS*	52.35 ± 0.19	214.47 ± 1.07	166.33 ± 0.92
TCOD*	19.42 ± 0.47	277.12 ± 0.59	219.57 ± 1.23
SCOD*	4.86 ± 0.13	15.08 ± 0.09	53.66 ± 0.07
Ammonia-N*	0.89 ± 0.03	2.66 ± 0.07	2.96 ± 0.03
Cellulose**	91.49 ± 0.49	307.19 ± 1.83	254.34 ± 0.59
Hemi-cellulose**	57.38 ± 0.29	227.61 ± 0.37	182.99 ± 0.70
Lignin**	51.73 ± 0.88	190.04 ± 1.07	147.23 ± 0.73
Total VFA*	1.06 ± 0.55	3.41 ± 0.39	4.56 ± 0.41
Carbon (wt%)	ND	46.11 ± 0.17	ND
Oxygen (wt%)	ND	31.08 ± 0.52	ND
Hydrogen (wt%)	ND	7.39 ± 0.48	ND
Nitrogen (wt%)	ND	6.22 ± 0.08	ND
Sulfur (wt%)	ND	0.96 ± 0.07	ND
TMY (mL g^− 1^VS)		531.87 ± 7.54	

*Wet weight (g/kg); **dry weight (g/kg).

### The freezing and thawing pretreatment

The freezing and thawing pretreatment of feedstock was carried out during the winter season of 2020–2021 in Beijing, China. The fresh CM was packed in plastic buckets and placed outdoors in the laboratory courtyard for freezing and thawing. The total time of pretreatment was 20 days based on a previous study^12^. Throughout the pretreatment period automatic temperature recorder was installed on site to record the daily and hourly temperature.

### Experimental setup

Two parallel batch experiments were designed to test the biomethane potential of RCM and PCM feedstocks with an automatic methane potential test system (AMPTS^®^, BPC Sweden). Each reactor volume was 500 mL with a 400 mL working volume. The reactors were operated at 5%, 10%, 15% and 20% TS in triplicate with substrate to inoculum ratio of 1:1 based on the previous study^10^. All reactors were flushed with nitrogen gas for about 2 min then airtight with rubber stoppers. The mechanical agitation off-time and on-time intervals for each reactor were 60s and 30s respectively, in the clockwise rotation to ensure that each reactor is operating under optimum mass transfer conditions. The operating temperature was in the mesophilic temperature range. The total duration of AD experiments was 45 days.

### Theoretical methane potential and biodegradability

The maximum methane potential that can be assumed by specific feedstock, was calculated based on elemental analysis of substrate with the assumption of a homogenized mixture with ideal microbial conditions and complete conversion of organic materials at constant temperature and pressure according to Buswell formula^13^. The method was based on elemental composition. Boyle modified the chemical reaction of Buswell and Mueller^13^ and included nitrogen and sulfur to obtain the fraction of ammonia and hydrogen sulfide in the produced biogas. Thus, the maximum methane potential of CM was calculated according to Eqs. (1), (2), and is expressed in mL CH4 g^− 1^VS^14,15^.

1 $$\begin{aligned} &C_{a}{H}_{b}{O}_{c}{N}_{d}{S}_{e}+\left(a-\frac{b}{4}-\frac{c}{2}+\frac{3d}{4}+\frac{e}{2}\right){H}_{2}O\to\left(\frac{a}{2}+\frac{b}{8}-\frac{c}{4}-\frac{3d}{8}-\frac{e}{4}\right){CH}_{4}\\ &\quad+\left(\frac{a}{2}-\frac{b}{8}+\frac{c}{4}+\frac{3d}{8}+\frac{e}{4}\right){CO}_{2}+d{NH}_{3}+e{H}_{2}S\end{aligned}$$

The reaction is balanced automatically and could easily be applied to any input of known relative ratios of C, H, O, N and S. This model assumes these elements are only the components of the feedstock.

2 $$TMY=\frac{(\frac{a}{2}+\frac{b}{8}-\frac{c}{4}-\frac{3d}{8}-\frac{e}{4})\cdot 22400}{(12a+b+16c+14d+32e)}$$

where *TMY* is the theoretical maximum methane potential and *a* is the number of carbon atoms, *b* is hydrogen atoms, *c* is oxygen atoms, *d* is nitrogen atoms, and *e* is suplhur atoms.

The anaerobic biodegradation was calculated with the following equation (Eq. 3)^16^.

3 $${BD}_{{CH}_{4}}=\frac{BMP}{{TMY}_{ele}}\times100$$

The daily and cumulative methane yield of each reactor was measured in real-time measurement for 45 days. The gas volume and flow rate were standardized to zero moisture content, 0 °C temperature and 1 atm pressure for all reactors.

### Kinetics models

The hydrolysis is considered as the rate-limiting step of HSAD that controls the entire process. Hence, for the determination of hydrolysis and the cumulative methane yield potential of PCM and RCM, the first order kinetic model was used^17^.

4 $$M\left(t\right)={P}_{m}(1-{e}^{\left(-kt\right)})$$

where, *M(t)* is cumulative methane yield (mL CH_4_/g VS_added_) in relation to digestion time *t* (days); *Pm* is the maximum methane potential of the feedstock (mL CH_4_/g VS_added_); *K* is hydrolysis rate constant (1/day) and *t* is digestion time (days). The first order model is categorized as a classical kinetics model for the simulation of different feed-stocks in AD process. Gompertz proposed a model that provides knowledge about the cell growth rate and lag phase duration as variables. It explained very well the microbial community dynamics and methane yield^18^.

5 $$M\left(t\right)={P}_{m}\times\text{exp}(-\text{exp}\left(\frac{{R}_{m}\times{exp}\left(1\right)}{{P}_{m}}\right)\left(\lambda-t\right)+1)$$

where, *M(t)* is the cumulative methane yield (mL CH_4_ /g VS_added_) regarding time t, *Pm* is the maximum methane yield (mL CH_4_/g VS_added_.d), *t* is digestion time (days), *Rm* is the methane yield rate (mL CH_4_/g VS_added_), *λ* is lag phase time (days) and *e* is Euler’s function i.e. the base of natural logarithms with a value of 2.71828. The parameters such as *P*_*m*_, *R*_*m*_, *k*, and *λ* were calculated by the nonlinear least-square regression analysis in SPSS statistics v25. The standard error (SE) root mean square error (RMSE), and correlation coefficient (R^2^) from each model were also acquired. The origin software (origin2018, OriginLab Corporation) was used for data analysis and graphing of the experimental methane yield and the predicted methane from SPSS statistical data.

### Biomass sampling

After 45 days of digestion, a total of 8 representative samples were collected from RCM and PCM digesters comprising 5%, 10%, 15% and 20% solid concentration. The sampling was carried out in 50 mL plastic tubes (Ep-5000, Wuhan Servicebio Technology Co., Ltd) and immediately stored in a refrigerator at a 4 °C temperature. Through an ice-box, refrigerated samples were shifted in an ultra-low temperature (-80 °C) freezer prior to the DNA extraction.

#### DNA extraction

In order to get a homogenized representation, the extraction and sequencing were achieved in triplicate. Before the DNA extraction, the samples were deiced, grazed and blended at room temperature with an aim to achieve uniformity. The QIAamp PowerFecal Pro DNA kit (Mo Bio Laboratories, CA 92010, US) was used for the higher yield of DNA from digestate samples.

#### 16s rRNA gene sequencing

After DNA extraction, the primers were acquired. For fishing DNA sequencing, sequencing adapters were used in the end region. The sequencing library was constructed by purifying, quantifying and homogenizing the resultant product. Illumina HiSeq 2500 was used to sequence Qualified libraries. Agarose gel (1%) was added to the electrophoresis apparatus (DYCZ20F, Liuyi Biotech Ltd. Beijing) and was used for the examination of DNA integrity. The PCR was accomplished by TransGen-AP221-02TransStart^®^Fastpfu DNA polymerase. To ensure optimum reaction conditions the following thermo-cycler was added: denaturation step: 95 °C for 3 min, followed by 35 cycles at 95 °C (30 s), 55 °C (15 s) then 72 °C (45 s) and 72 °C for 10 min, finally 10 °C for 5 min for annealing step.

For quality insurance, Agarose Gel Electrophoresis (agarose gel 2%) was used before rapid and high output Illumina HiSeq 2500 (USA). The FLASH (Fast Length Adjustment of SHort reads) (v1.2.11) was used to make and stitch the paired-end reads from individual sample to generate the raw tags from the subsequent stitching data. The Trimmomatic v0.33 software was used for the filtration of raw tags and chimera detection was amplified using UCHIME v4.2.40. By using USEARCH-UPARSE (v.10.0.240), the optimized sequences were grouped into operational taxonomic units (OTU). The Ribosomal Database Project (RDP) Classifier version 2.2 and Greengenes version 13.8 were used for the OTU representativeness (confidence threshold 0.8). The rarefied OTU table was used to measure the alpha diversity metrics while the beta diversity analysis was performed through QIIME suite of tools (Version 1.8.0).

### Other analytical methods

The determination method used to determine total chemical oxygen demand (TCOD) and soluble chemical oxygen demand (SCOD) was based on the fast digestion spectrophotometric technique. The ammonia-N concentration was analyzed by the Nessler spectrophotometric method with the equipment and reagents from Shanghai Lianhua, China with standard protocol. The protein content in RCM, PCM and digestate samples was measured by Bicinchoninic acid (BCA) method in agreement with the protocols described by Nanjing Jiancheng Biotechnol. Co. Ltd, China). The volatile fatty acids (VFAs) were measured by gas chromatography coupled via a flame ionisation detector through Shimadzu 14 C-GC (Shimadzu, Japan). The hemicellulose, cellulose and lignin contents were measured by the method used by Yuwan and Wenyu^19^. The Vario EL-III Cube Elemental analyzer (GmbH, 2003) was used to determine elemental composition (Carbon, nitrogen, hydrogen, and sulfur) of RCM and PCM samples.

## Results and discussions

### Pretreatment performance

**Fig. 1:**
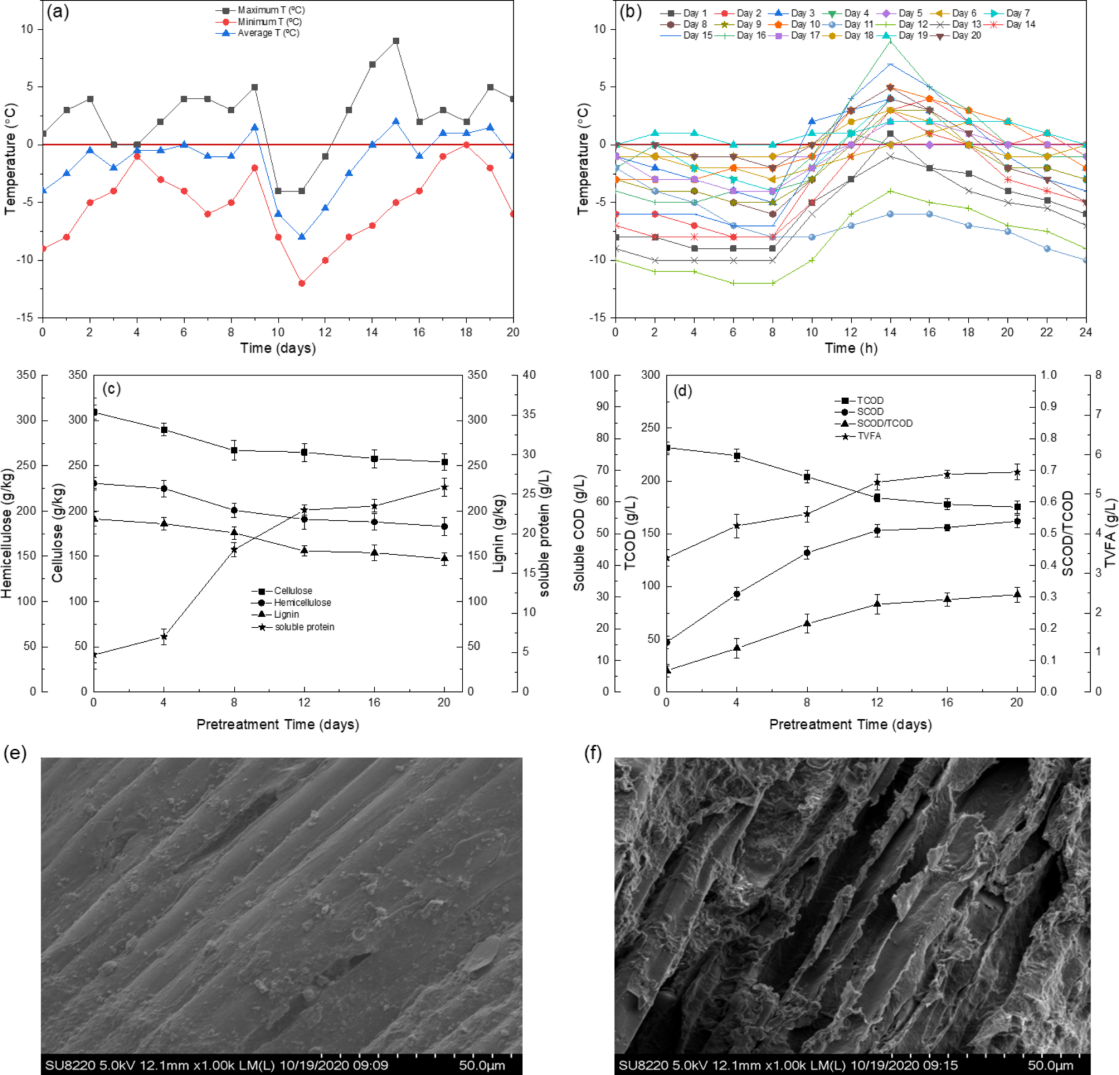
Pretreatment temperature (**a**) maximum, minimum and average temperature, (**b**) 24-hour fluctuation in temperature, (**c**) the variation in cellulose, hemicellulose, lignin and soluble protein, (**d**) change in TCOD, SCOD, SCOD/TCOD and TVFAs during pretreatment, (**e**) SEM image of untreated CM and (**f**) SEM of pretreated CM.

The effect of seasonal freezing and thawing pretreatment on undiluted CM was investigated (Fig. 1). During the winter season, Beijing’s temperature falls below 0 °C which provides very suitable conditions for the natural freezing and thawing process. The lowest and highest temperatures recorded during this pretreatment period were − 12 °C and 9 °C, respectively (Fig. 1a,b). The freezing was observed usually at night (8:00 pm and thawing time was morning 8:00 am) when the temperature rose enough. The average temperature was seen between − 8 °C and 4.0 °C. Furthermore, the almost everyday temperature was observed below and above 0 °C, which was desirable for the freezing and thawing process. During the pretreatment period, cellulose, hemicellulose, lignin, protein, TCOD, SCOD and TVFA were measured consecutively. The SEM analysis was performed before and after pretreatment. The pretreatment period of 20 days was selected based on a previous study^12^, however in our experiment we found that the effective pretreatment period for CM could be 10–12 days because a minor change was observed in physicochemical properties in the coming days.

The RCM had a high content of lignocellulosic substances, for example, cellulose 309.32 g/kg, hemicellulose 230.73 g/kg and lignin 191.29 g/kg. After pretreatment, the reduction in cellulose content was 17.8%, hemicellulose was 16.3% and lignin was 13.3%, as shown in Fig. 1c. The decline of lignocellulosic contents could be attributed to the rupturing of the plasma membrane of plant cells (manure mainly contains residues from fodder). This happened when at extremely low temperatures, large amounts of intracellular ice crystals were formed which increased the mechanical stress on the plasma membrane due to dehydration and during thawing the melting of ice seriously disrupted the structure of the cell due to hydrostatic pressure^20^.

The SCOD concentration increased in relation to pretreatment time, as illustrated in Fig. 1d. The initial SCOD concentration was 15.73 g/L and it increased to 53.94 g/L due to freezing and thawing disintegration of particulate COD and other organics. The SCOD concentration of PCM was 3.43 folds higher than RCM. The solubilization efficiency and SCOD/TCOD ratios are the basic tools to measure the hydrolysis rate and organic matter dissolution during pretreatment. The ratio between SCOD/TCOD increased from 0.068 to 0.308 while 75% solubilization efficiency was enhanced. Similar results were found in a study conducted by Xue et al.^21^, in the thermal pretreatment of high solid sludge, the COD solubilization was increased from 4.5 to 41.1% at 90 °C.

During the pretreatment, the release of SCOD is considered an essential indicator for evaluating the hydrolysis efficiency of pretreatment techniques^22^. To generate high SCOD concentration, long-term harsh pretreatments have been adopted that may release some highly recalcitrant soluble organics during pretreatment e.g. inhibitory/toxic intermediates such as long-chain VFAs, high ammonia-N etc., high-molecular polymer which can suppressed AD process. During freezing and thawing pretreatment there was no elevated ammonia-N or high inhibitory VFA production observed hence, indicating it a suitable pretreatment of CM for AD.

The soluble protein concentration increased by 5.5 folds (4.71 g/L to 25.89 g/L) after pretreatment as shown in Fig. 1c. The degradation of COD enhanced TVFA concentration (Fig. 1d hence, the accumulation of TVFA lowered the pH of the substrate. These results are in line with previous findings in food waste^23^ and in activated and alum sludges^24^. The moisture content of CM without any dilution were approximately 75–85%, which provides ideal moisture for the freezing and thawing phenomenon. Freezing can seize the microbial activity because it blocks pores and reduces the substrate and water availability hence causing starvation for microbes even desiccation and death^25^.

The subsequent thawing minimizes the risk of microbial fatality even recovery rate was seen as high as the initial level^26^. This might be due to the adaptability of microorganisms towards extremely low temperatures in continuous stress hence resulting in enhanced biodegradability and decomposition of manure. The freezing and thawing enabled microbes to attach to the surface to grow and reproduce^27^. Hence, they played a significant role in lignocellulosic conversion during pretreatment and enhanced biogas production during HSAD without alteration. Figure 1e and f shows the morphological characteristics of CM before and after pretreatment. The SEM analysis of PCM and RCM clearly provided evidence of change in the physical structure after the pretreatment process (Fig. 1e,f). The linear rigid and biopolymeric structure with a contiguous, flat and smooth surface was deflated and distorted causing severe cracks in the surface. Hence, rendering it more susceptible to enzymatic attack during hydrolysis.

Similar results were found in previous studies when corn stalks, plant residues, anaerobic sludge and garden waste were subjected to natural freezing-thawing pretreatment^12,28–30^. When organic wastes were subjected to freezing, the ice crystals were formed in the extracellular space that hindered the solutes movement, leading to an increase in the concentration of solute at the interface between ice and water^31^. The accumulation of extracellular substances allows ice to grow as a finger-like structure at the microscale in the cytoplasmic region of the cell. Ragoonanan and Vishard^32^ found that these needles-like ice crystals present inside the cell create a chemical potential difference between the hypertonic extracellular substances and the supercooled intracellular solution and hence provide a force to efflux water from cells, which causes the cell to shrink, dehydrate, or even destroy.

### Biomethane yield

**Fig. 2:**
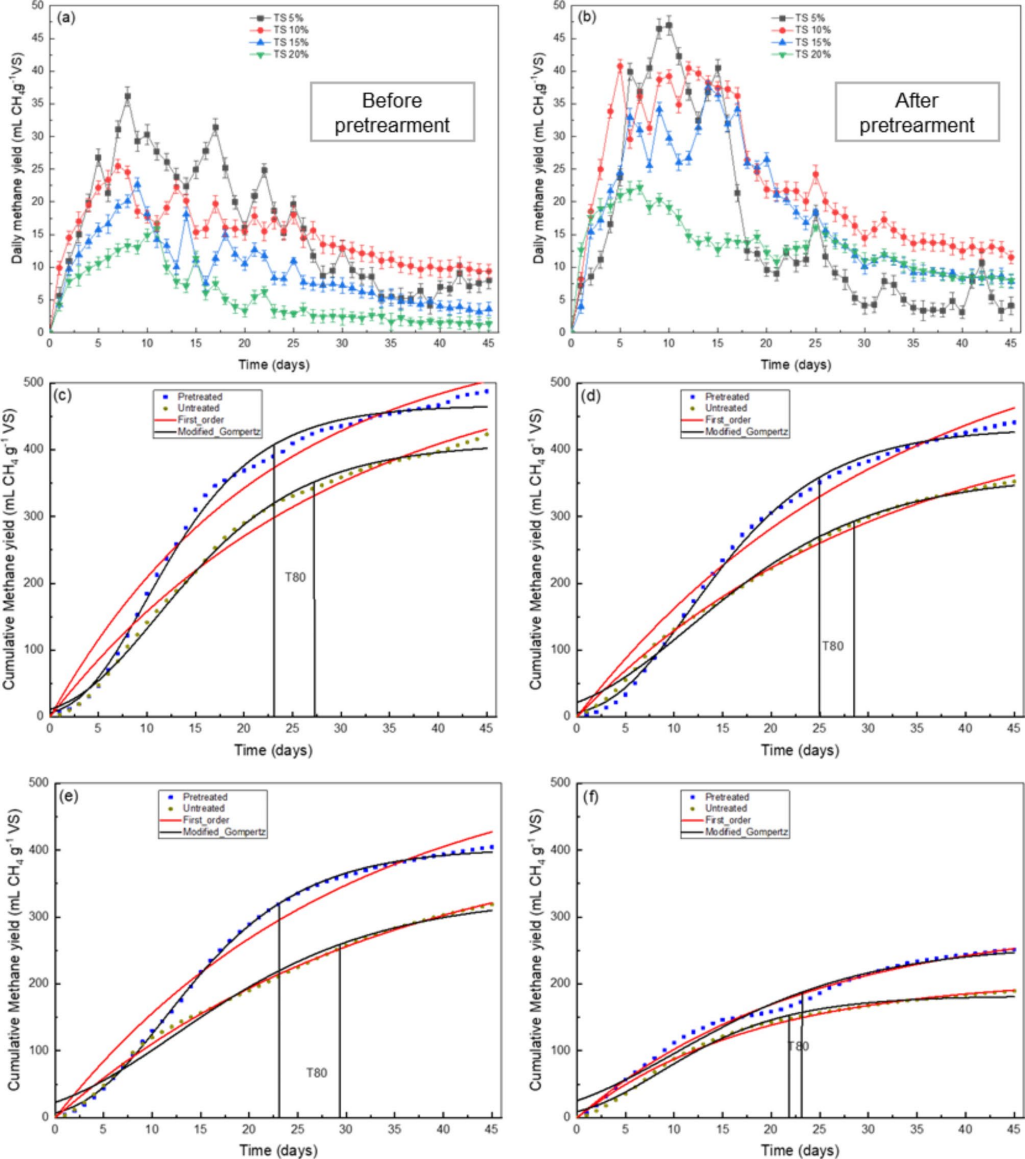
Daily methane yield at different solid content (mL CH_4_ g^− 1^VS): (**a**) untreated CM, (**b**) pretreated CM; Cumulative methane yield graph and fitting the curves of first order kinetics (red lines) and modified Gompertz model (black lines): (**c**) TS 5%, (**d**) TS 10%, (**e**) TS 15%, (**f**) TS 20%.

The biomethane yield of RCM and PCM digesters with increasing solid content was investigated and results are shown in Fig. 2a–f. These results revealed that methane yield was enhanced profoundly after pretreatment. The peak values explicate the maximum daily methane yield (DMY) rate while digestion time is equivalent to peak time denoting the DMY rate. Among the peaks of RCM, the first peak of DMY reached 27 mL on 6th day, 25 mL on 7th day, 22 mL on the 9th day and 14mL on the 9th day in TS 5%, TS 10%, TS 15% and TS 20% digester, respectively. For PCM, the first peak was raised to 40 mL on day 4, 41 mL on day 3, 34 mL on day 5 and 23 mL on day 7 in TS 5%, TS 10%, TS 15% and TS 20% digesters, respectively.

The time period of the first peak in PCM was shorter than RCM and the second peak for pretreated TS 5% digester appeared at day 6, which was 2 days earlier than RCM, indicating that pretreatment has improved the hydrolysis rate significantly. Same results were also found for TS 10%, TS 15% and TS 20% and the second peak was found 4d, 6d and 2d earlier, respectively. These findings revealed that the pretreatment has remarkably enhanced the digestibility of PCM digesters. The DMY efficiency of PCM was 1.05, 1.81, 2.78, and 1.73 folds higher than RCM at TS 5%, 10% 15% and 20%, respectively.

The maximum cumulative methane yield (CMY) (487 mL g^− 1^VS) was found in TS 5% digester followed by 10% TS (441 mL g^− 1^VS), 15% TS (405 mL g^− 1^VS) and 20% TS (251 mL g^− 1^VS) in PCM. The CMY rate of PCM was observed 13%, 20%, 21% and 25% more than RCM at TS 5%, 10%, 15% and 20% respectively. An inverse relationship was observed between TS content and biomethane yield indicating digester complexity with increasing solid concentration. The methane yield of freezing and thawing pretreated CM (present study) was 6% higher than ultrasound pretreated and crude glycerine co-digested CM at TS 8.6^33^ and it was 35% higher than acid (2% of HCl) and alkali (10% of NaOH) pretreated CM^3^ digested at a mesophilic temperature between 5 and 8% TS range.

Biomethane yield of freezing and thawing pretreated CM between 10 and 15% TS was 87% higher than mechanically pretreated (shredding, mixing, and blending) CM^4^, 472.4% higher than Acid (1% H_2_SO_4_) pretreated CM^34^, 94.2% higher than alkaline thermal hydrolysed (2% NaOH, 160 °C) CM^35^ and 142.2% higher than thermally hydrolysed (160 ℃, 30 min) CM^36^. Hence, natural freezing and thawing pretreatment could prove a better pretreatment technique over, physical, chemical, or biological pretreatment in terms of higher biomethane yield.

### Process parameters

**Fig. 3:**
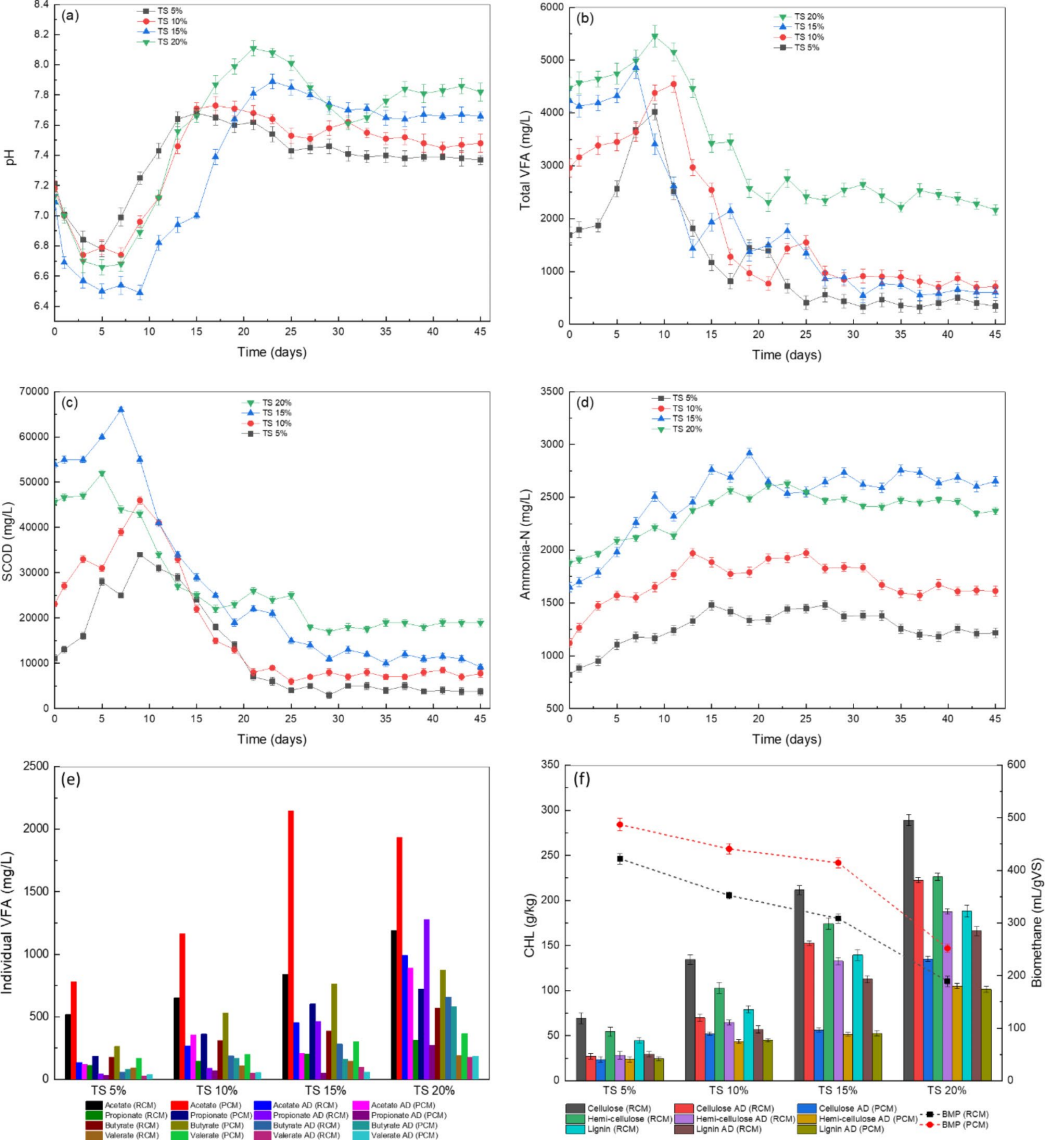
(**a**) Change in pH, (**b**) total VFA, (**c**) SCOD, (**d**) ammonia-N, (**e**) individual VFA and (**f**) lignocellulosic contents during HSAD process.

The pH is considered one of the most important parameters of the AD process. It is extremely sensitive to VFA and buffering capacity, especially during the HSAD process when TS is significantly higher. The most suitable pH for fermentative bacteria and methanogenic archaea lies in the range of 5.0 to 8.5 and 6.5 to 8.2, respectively^37^. Freezing and thawing pretreatment significantly lowered the pH in PCM due to the dissolution of organics. Figure 3a shows the variation in pH during the HSAD process. A gradual decline in pH was observed in all digesters however remained over 6.5, showing the stability of the process.

The decline of pH is directly related to the degradation of carbohydrates, lipids, protein, and polysaccharides, consequently increasing VFA and other intermediate acids^38^.

Fatty acids are considered important intermediates for methanogenic archaea to produce biomethane. At the beginning of the AD process, the VFA were the principal organic compounds available for methanogens as substrate. Figure 3b and e depict the initial individual VFA concentrations in PCM and the change in total VFA during the AD process. The pretreatment of manure considerably enhanced VFA concentration. Acetate was the main constituent of VFA succeeded by butyrate, its concentration in PCM was found 0.78 g/L, 1.17 g/L, 2.15 g/L and 1.93 g/L at TS 5%, 10%, 15% and 20%, respectively. It was 51%, 78%, 155% and 62% higher than RCM at 5%TS, 10% TS, 15% TS and 20% TS, respectively. With the elapse of time, the VFA concentration was increased initially and then decreased. These findings are consistent with previous research^39^.

The VFA concentration was increased from day 1 to 9 at TS 5%, 1 to 11 at TS 10%, 1 to 7 at TS 15% and 1 to 13 at TS 20%, this was 2 days, 4 days, 9 days and 5 days earlier then RCM, respectively. The dissolution of VFA was observed faster in PCM with a steep slope in all reactors, as shown in Fig. 3b. Freezing and thawing pretreatment significantly reduced the inhibitory impact of VFA in all digesters by balancing the acidogenesis, acetogenesis and methanogenesis process. However, TS 20% digester achieved the highest concentration of VFA (5.73 g/L) on the 17th day together with elevated propionate (1.45 g/L) surprisingly 4.6 folds greater than the day first propionate concentration. The higher concentration of propionate (1,000 ~ 2,000 mg/L) is highly toxic to methanogens and capable to deteriorate digester performance^40^.

The SCOD concentration first increased and then decreased rapidly, clearly indicating that freezing and thawing pretreatment disintegrated long-chain complex organics into simple and easily biodegradable compounds as shown in Fig. 3c. The SCOD rising and declining rates in PCM were faster and higher. The SCOD steep of pretreated substrate reached its minimum 8 days, 10 days, 7 days and 13 days earlier than RCM at TS levels 5%, 10%, 15% and 20%, respectively. The COD removal efficiency of PCM was found 75%, 74%, 71% and 50% which was 7%, 10%, 24% and 21% higher than RCM at TS 5%, 10%, 15% and 20%, respectively. The COD removal rate was remarkably promoted at TS 15%. The primary cause might be the ideal hydrolysis and microbial consolidation, which enhanced the degradation and solubilization process.

With increasing TS, a gradual increase in ammonia-N concentration was observed in all digesters, as shown in Fig. 3d. The highest ammonia-N (2917 mg/L) was recorded on day 17th at 15% solid content which was in the range of the permissible limit (3300 mg/L) of mesophilic digesters. Westerholm et al.^41^ noted that at 3300 mg/L ammonia-N concentration and pH 7.9, the biomethane yield was optimum in the mesophilic digester. However, biomethane yield was decreased to 50% when ammonia-N concentration went up to 5500 mg/L. In this study, ammonia-N concentration in all digesters was found in the optimum range. Hence, no ammonia inhibition was observed during HSAD.

### Biodegradability

The cellulose, hemicellulose and lignin (CHL) were the main components of CM. Due to their low hydrolysis rate and complex degradability, they were known as poorly biodegradable organics. The bioconversion of lignocellulosic components of CM into methane is only possible through the pretreatment of the substrate before AD and a strong cooperation of robust mixed microbial communities during AD process^42^. As illustrated in Fig. 3f, In PCM digesters the reduction rate of cellulose was 66%, 61%, 73% and 53%, which was 14%, 25%, 63%, and 39% higher than RCM digesters at 5%TS, 10% TS, 15% TS and 20% TS respectively. The reduction rate of hemicellulose in PCM digesters was 16%, 33%, 61% and 44% higher than in RCM digesters while the reduction rate of lignin was 17%, 21%, 53% and 39% higher than in RCM at 5%, 10%, 15% and 20% solid concentration, respectively.

The freezing and thawing pretreatment mainly disrupted the lignocellulosic matrix by rupturing the cell wall, and plasma membrane and then breaking glycosidic linkage, resulting in the high conversion of polysaccharides in a short time and consequently higher methane production. The peak reduction was seen at TS 15% for cellulose (64%), hemicellulose (57%) and lignin (43%) which was 2.7, 2.5, and 2.1 folds higher than RCM at TS 15%, respectively. These results were consistent with previous studies^12^. The degradation of cellulose was higher than hemicellulose followed by lignin (cellulose > hemicellulose > lignin) during both pretreatment and AD process indicating that the biomethane and CO_2_ mainly originated during the hydrolysis of cellulose and hemicellulose.

The biodegradability of PCM was higher than the RCM and it decreased with increasing TS in both PCM and RCM digesters. The decrease in biodegradability with increasing TS could be attributed to the complexity of the system due to high OLR, low moisture content and less available sites for microbial consortium within the substrate. However, in PCM digesters, the biodegradability was higher due to freezing and thawing pretreatment that enhanced the hydrolysis of lignocellulosic substances due to certain physicochemical and biological changes. The highest methane yield of 487 mL g^− 1^VS was achieved in the PCM reactor at 5% TS which was close to the TMY of CM (531.87 mL g^− 1^VS) calculated from Eq. 1. The biodegradability of PCM digesters at 5%, 10%, 15% and 20% TS was 91.61%, 82.88%, 76.06% and 47.23% that was 15.21%, 25.16%, 26.95% and 32.63% higher than the RCM digesters respectively. The freezing and thawing pretreatment significantly enhanced the biodegradability of hardly biodegradable components of CM.

### Kinetics parameters

The CMY rate of freezing and thawing pretreated CM fitted well with the modified Gompertz model (Fig. 2; Table 2) with R^2^ of 0.996, 0.997 and 0.999 for TS 5%, 10% and 15%, respectively, indicating that hydrolysis rate was higher after pretreatment. The reactor startup time period is very important for the enhanced AD process. The calculated lag-phase time (λ) of freezing and thawing PCM was 3.18 days, 3.62 days, 3.21 days and 4.81 days that was 4.2%, 10.3%, 34.6% and 23.3% shorter than RCM at TS 5%, 10%, 15% and 20%, respectively. The smaller λ value in PCM digesters predicts the fast and higher degradation rate of organics in comparison with RCM digesters. In contrast, the longer λ was due to a higher percentage of crude protein and lingo-cellulosic substances in RCM that lowered the degradation rate. The shortest λ (3.21 days) and the highest R^2^ value (0.999) were observed at 15% TS indicating that poorly and readily biodegradable organics were utilized due to balanced process parameters and enhanced microbial activity, consequently higher biomethane yield. These findings are in agreement with the previous studies^12^. At 20% TS the CMY of PCM was fitted well with first order model having 0.993 R^2^ value and 0.032 d^− 1^ k value. This indicates that the degradation rate of hardly degradable compounds was poor due to higher viscosity and compact system^43^. The T80 (time period for 80% of total biogas production) calculated in PCM was 4 days, 3 days and 6 days earlier than RCM at 5% TS, 10% TS and 15% TS respectively, as shown in Fig. 2c–f. The shorter T80 value obtained from the 20% TS digester was probably because of low CMY comparatively.

**Table 2:** The kinetics parameters evaluated from first order kinetic model and modified Gompertz model during HSAD of CM.

Condition	TS (%)	EMY (mL/gVS)	First order kinetics model					Modified Gompertz model	
			P (mL/gVS)	K (1/d)	R^2^	P (mL/gVS)		λ (d)	R^2^
Pretreated CM	5	487.26	580.48	0.045	0.968	466.50		3.18	0.996
	10	440.83	623.19	0.030	0.973	432.88		3.62	0.997
	15	404.55	548.87	0.033	0.975	403.12		3.21	0.999
	20	251.22	297.31	0.032	0.993	259.87		4.81	0.985
Untreated CM	5	422.95	551.45	0.034	0.983	409.76		3.46	0.997
	10	352.22	476	0.032	0.994	363.89		5.45	0.994
	15	318.66	441.91	0.029	0.997	332.46		6.205	0.988
	20	189.40	208.63	0.024	0.998	182.46		9.739	0.985

EMY: Experimental methane yield, P: simulated maximum CMY, K: biodegradability rate constant, R^2^: correlation coefficient, λ: Bacterial growth lag time.

### Microbial community

#### Microbial community diversity and richness

In order to deep understanding of the digester’s stability during the HSAD process, the specific function and performance of each microbial group was correlated with increasing solid content. The microbial population in a certain digester was different from others showing the sensitivity of microbes during prevailing treatment conditions^44^. Table 3 shows the microbial diversity and richness in PCM and RCM digestate. Less diversity was observed in the archaeal community in comparison with the bacterial community. The higher value of the coverage index (0.99.9%) is anticipating the representativeness of each sample. The greater ACE and Chao1 values of PCM samples indicated that freezing and thawing pretreatment did not affect the microbial community dynamics and during HSAD the bacterial and archaeal diversity significantly enhanced due to ideal and favorable conditions. The Shannon index of archaea increased with increasing solid content in PCM digestate. At TS 10% and 15% Shannon index was 2.63 and 4.69 which was 2.2 and 2.5 folds higher than RCM, respectively. Ideally, at higher TS levels, the diverse archaeal and bacterial communities are capable of maintaining comprehensive stability of biogas reactors by empowering continuous methanogenesis even though experiencing stress. Thus, freezing and thawing pretreatment of manure could be a possible physical pretreatment technique to enhance biomethane yield even at higher TS without any inhibition or stress to methanogens.

**Table 3:** Microbial community richness and diversity indexes before and after pretreatment under different solid concentration.

Treatment	Sample ID	OTU	ACE	Chao1	Simpson	Shannon	Coverage
Untreated CM	BacTS5	469	521.23	539.65	0.038	4.137	0.9948
	BacTS10	411	465.79	481.43	0.040	4.100	0.9949
	BacTS15	376	395.35	406.53	0.064	4.109	0.9984
	BacTS20	323	388.48	400.02	0.069	3.551	0.9949
	ArcTS5	160	179.32	176.71	0.200	1.106	0.9996
	ArcTS10	153	171.86	168.78	0.167	1.194	0.9996
	ArcTS15	163	180.30	179.72	0.263	1.845	0.9995
	ArcTS20	158	171.68	181.00	0.207	2.136	0.9995
Pretreated CM	BacTS5	647	666.58	658.08	0.034	4.401	0.9992
	BacTS10	617	650.49	653.49	0.029	4.575	0.9987
	BacTS15	575	577.93	577.50	0.027	4.873	0.9998
	BacTS20	569	582.20	577.36	0.024	4.571	0.9995
	ArcTS5	351	367.42	365.87	0.108	2.189	0.9994
	ArcTS10	269	353.18	364.35	0.518	2.636	0.9985
	ArcTS15	581	581.15	581	0.052	4.689	1
	ArcTS20	173	255.18	252.44	0.726	2.832	0.999

#### Bacterial community

**Fig. 4:**
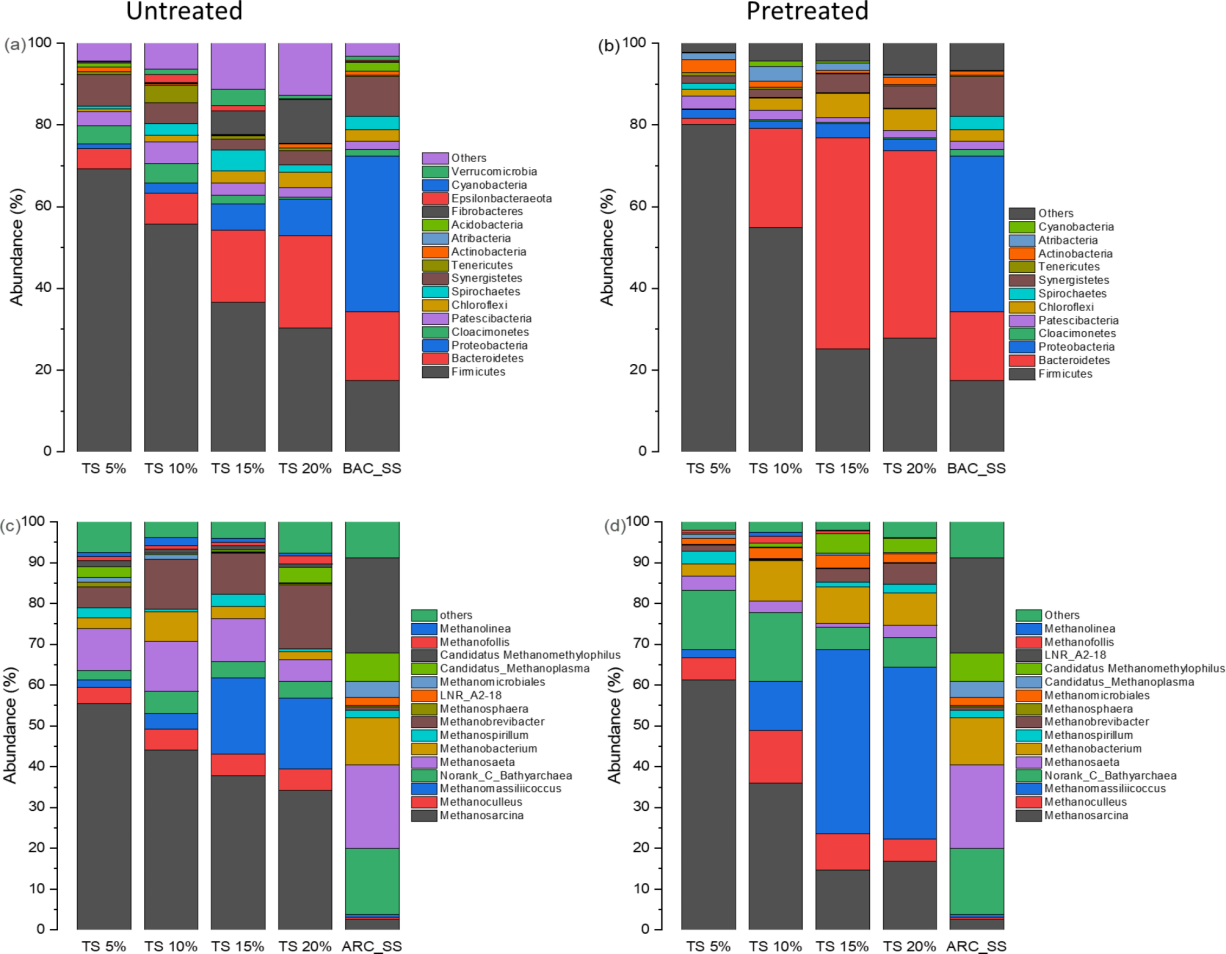
(**a**, **b**) bacterial community structure of untreated and pretreated CM and seeding sludge at the phylum level, (**c**, **d**) archaeal community structure of untreated and pretreated CM and seeding sludge at the genus level.

A total of 367 genera of bacterial community were observed in PCM digesters, while 237 genera were detected in RCM digesters. The 6 major bacterial phyla involved in the fermentation of manure were *Firmicutes*, *Proteobacteria*,* Bacteroidetes*, *Chloroflexi*, *Cloacimonetes*, and *Synergistetes* as shown in Fig. 4a and b.

*Firmicutes* proportion was 69%, 55%, 36%, and 30% in RCM while 80%, 54%, 25% and 27% in PCM digesters at TS 5%, 10%, 15% and 20% respectively.

The genus *Caldicoprobacter* among the phylum *Firmicutes*, was the predominant bacteria in RCM digesters and its proportion increased to 17%, 32%, 29% and 62% with increasing TS right after *Fastidiosipila*, *Sedimentibacter*,* Christensenellaceae_R-7_group*, *Herbinix* and *Ruminiclostridium*, respectively. While in PCM reactors, the most abundant genus was *Fastidiosipila* followed by *Romboutsia*, *Caldicoprobacter*, *Sedimentibacter*, *Ruminiclostridium* and *Paeniclostridium*. The *Caldicoprobacter* most likely degrades polysaccharides to produce acetate, lactate, ethanol, H_2_ and CO_2_^45^. The *Fastidiosipila* is assigned as proteolytic bacteria engaged in protein degradation in mesophilic digesters as described in previous studies^46^. The second abundant genera within the *Firmicutes* in PCM digesters was *Romboutsia*, these bacteria are responsible for the degradation of fructose, sucrose, glucose and produce acetate, lactate, H_2,_ and CO_2_ during AD^47^.

The *Bacteroidetes* relative abundance was increased with increasing solid content. Its proportion in PCM digesters was observed 1.5%, 24%, 51% and 45% at TS 5%, 10%, 15% and 20% that was − 0.26, 1.9, 3 and 2 folds higher than RCM digesters, respectively as shown in Fig. 4a and b respectively. The *Bacteroidetes* are proteolytic bacteria involved in degrading various proteins and carbohydrates to VFAs and ammonia^45^. The *Ruminofilibacter*,* DMER64*, *Fermentimonas*, *uncultured_bacterium_f_ST-12K33* and *VadinBC27* were the dominant genera among the phylum *Bacteroidetes*.

The *Ruminofilibacter* is closely related to manure fermentation^48^ and the higher proportion of this genus explains well the enhanced hydrolysis rate and methane yield in PCM digesters.

The bacteria present within *Proteobacteria* phylum has the ability to degrade protein and sugars in manure. The *Alpha Proteobacteria* was the most dominant class followed by *Beta* and *Delta Proteobacteria* in both RCM and PCM digesters. The *Alpha*,* Beta*, and *Gamma Proteobacteria* are capable of utilizing acetate, propionate, butyrate and glucose during AD process^10^ .

The *Chloroflexi* are capable of degrading hardly degradable organics^49^. Their proportion was higher in PCM digesters (4%, 5%, 9% and 6% at solid concentration of 5%, 10%, 15% and 20%, respectively), showing higher microbial activity in freezing and thawing pretreated digestate. The *Anaerolineaceae* was the dominant family with a relative proportion of 19% at TS 5%, 35% at TS 10%, 60% at TS 15% and 58% at 20% solid content. The bacteria belong to *the Anaerolineaceae* family have been distinguished as chemoheterotrophic anaerobic fermenters involved in the degradation of carbohydrates, amino acid and peptides-rich proteinaceous substrates to produce acetate, propionate, H_2_ and ethanol.

#### Archaeal community

The phylum *Euryarchaeota* was the most dominant archaeal phylum detected in RCM and PCM digesters along with a small portion of phylum *Crenarchaeota*. At low TS concentration the acetoclastic methanogenic pathway was found the predominant methanogenic pathway adding > 70% of the total biomethane. *Methanosarcina* and *Methanosaeta* are the two well-known acetoclastic methanogens but interestingly, they could hardly be found dominant in a digester simultaneously^50^. As it could be evidenced from Fig. 4c and d that *Methanosarcina* was overawed *Methanosaeta* in all digesters. During fermentation, the *Methanosaeta* could rapidly flourish at low acetate pressure due to its smaller values of *K*_*S*_ (half-saturation coefficient) and *µmax* (maximum specific growth rate). However, higher acetate concentration could endorse the proportion of *Methanosarcina* in the digester because of its higher *K*_*S*_ and *µmax* values that enable it to absorb high acetate concentration competently^51^.

Freezing and thawing pretreatment significantly increased acetate concentration hence depressed the *Methanosaeta* and promote *Methanosarcina* at lower TS levels. However, at higher TS level the elevated concentration of ammonia and VFA was found toxic to *Methanosarcina* and *Methanosaeta* and henced decreased their proportion significantly^52^. As for morphology concerned, due to the thin filamented shape and widened surface area the *Methanosaeta* is more vulnerable and sensitive to higher concentration of VFA and ammonia. The *Methanosarcina* always remains in thick clustered form, making a natural shield against potential inhibitors and minimizing the risk of instability.

The archaea found in the class of *Methanomicrobia* can utilize CO_2_ as electron acceptor and H_2_ as an electron donor during the HSAD process. Some species are also capable of utilizing formate and secondary alcohol as alternative electron donors^50^. The *Methanoculleus*, *Methanofollis*,* Methanolinea* and *Methanospirillum* were the dominant hydrogenotrophic genera detected in the samples.

Among them, the *Methanoculleus* was a predominated genus in PCM and RCM digestates. Its abundance in PCM samples was 5.5% at TS 5%, 12.9% at TS 10%, 9.0% at TS 15% and 5.5% at TS 20% respectively, which was relatively higher than RCM digesters as can be seen in Fig. 4c and d. The *Methanoculleus* has the ability to utilize H_2_, CO_2_ or formate as a substrate to produce biomethane. A positive linear correlation was observed between ammonia-N and *Methanoculleus*, indicating its capability to survive even at elevated ammonia and VFA concentrations. When a pure cultured *Methanoculleus (i.e.*,* Methanoculleus bourgensis MS2*^*T*^*)* was bioaugmented to an ammonia-rich substrate in a CSTR reactor at elevated ammonia concentration, biomethane production was increased by 31.3% and a 5-fold increase in relative abundance of *Methanoculleus* was observed^53^. Hence, the bioaugmentation of *Methanoculleus* to a digester facing inhibition due to higher ammonia concentration could be a solution to stabilize the process and increase biomethane yield. As described earlier, the protein and lingo-cellulosic components were the major portion of CM, while during freezing and thawing, pretreatment ammonia-N concentration increased significantly promoting the *Methanoculleus* population during the AD process.

*Methanobacteria* are hydrogenotrophic methanogens that can utilize CO_2_ as an electron acceptor and H_2_, formate, secondary alcohol or CO serve as an electron donor. However, *Methansphaera* is the only genus of *Methanobacteria* that can reduce methanol with H_2_^50^. The proportion of *Methanobacterium* and *Methanobrevibacter* was higher in PCM and RCM digesters in comparison with the genus *Methanosphaera*. In PCM digesters, *Methanobacterium* can exist at higher ammonia and VFA concentrations and replace the acetoclastic methanogens (*Methanosarcina* and *Methanosaeta)* during the HSAD process along with increasing TS content. However, surprisingly the proportion of this archaeon decreased significantly between TS 15% and TS 20% in RCM digesters and one possible reason was the higher propionate concentration present in the system as described earlier^54^.

The genus *Methanobrevibacter* was found in abundant in RCM digesters with a proportion of 5.2%, 12.1%, 10.1% and 15.6% while in PCM digesters 1.38%, 0.28%, 3.33% and 5.12% at TS 5%, TS 10%, TS 15% and TS 20% digester respectively. The *Methanobrevibacter* is a highly acid-resistant genus and plays a very important role in system stability during low pH (5.0 -7.5)^50^. During HSAD of untreated manure, it was observed that a higher amount of VFA accumulated in the system resulting in lowering the pH and suppressing methanogenesis (TS 15% and TS 20%). The *Methanobrevibacter acididurans* presence shows the struggle to gain stability while the system was under pressure due to high VFA accumulation.

The class *Thermoplasmata* found in RCM and PCM digestates falls in the phylum *Euryarchaeota*. Because of the presence of unique methyl-coenzyme M reductase (MCR) genes, their methanogenic metabolic lifestyle was confirmed. Within the class *Thermoplasmata* the order *Methanomassiliicoccales* was found dominant and within the *Methanomassiliicoccales* the *Methanomassiliicoccus* was predominated genus in PCM and RCM digesters. Its proportion was 2.1%, 12.2%, 45.0% and 42.1% at TS 5%, 10%, 15% and 20% in PCM digesters while in RCM 1.8%, 3.7%, 18.7% and 17.4% respectively. *Methanomassiliicoccus* can utilize methylamines (mono-, di-, tri-) and dimethyl sulphide as substrates during HSAD and are also capable of reducing methanol with H_2_ during the AD process^55^.

Among archaeal methanogens, *Norank_C_Bathyarchaea* is unique due to its ability to perform complex metabolic pathways. A significant proportion of *Norank_C_Bathyarchaea* (phylum *Bathyarchaeota*) was detected in PCM digesters. It was observed in previous studies that *Norank_C_Bathyarchaea* showed better methanogenic performance in the absence of any potential inhibition^56^. The accumulation of VFA and ammonia in HSAD of untreated CM proved potential inhibitors for *Norank_C_Bathyarchaea*.

#### Methanogenesis

**Fig. 5:**
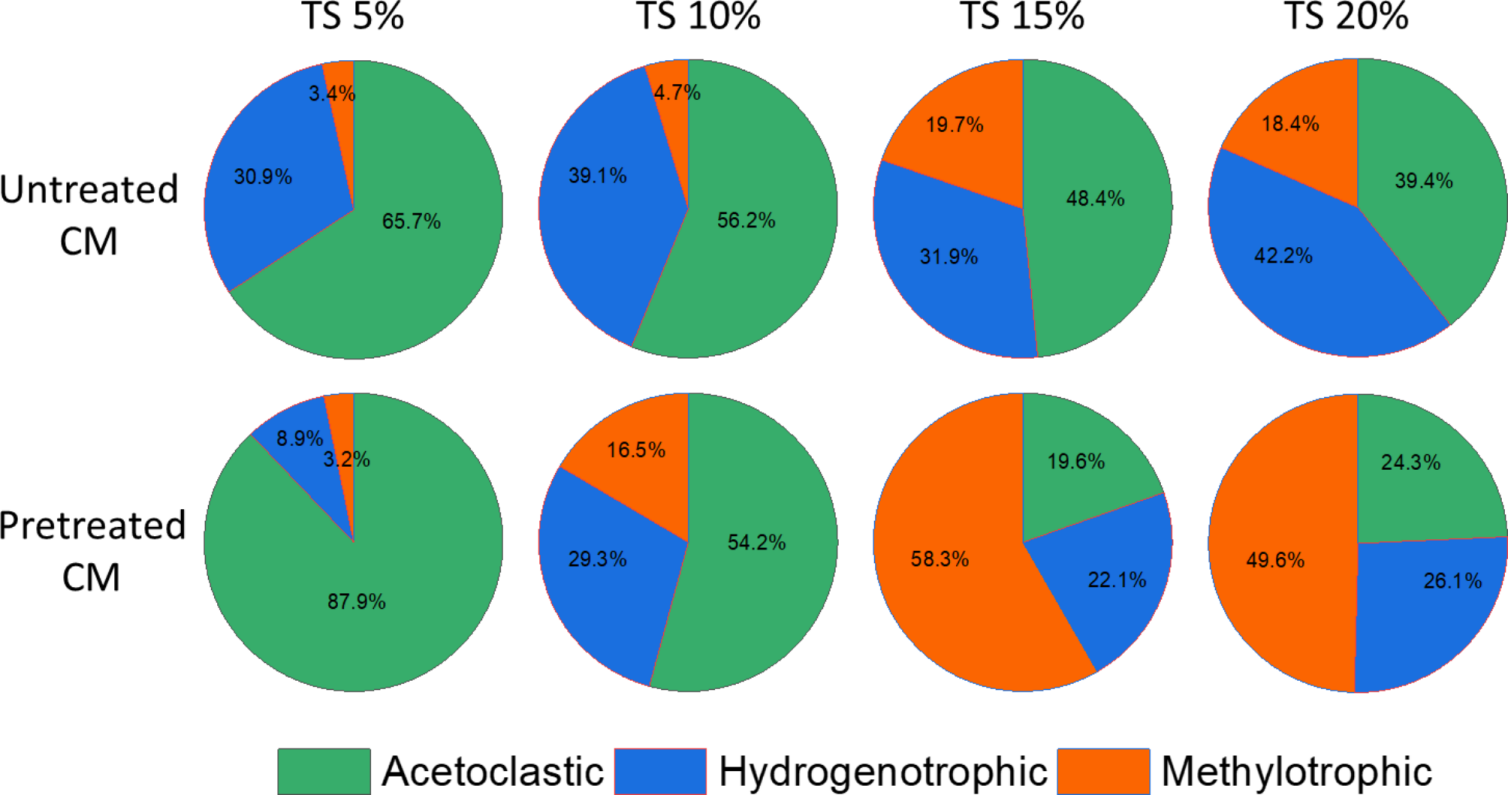
Main methanogenic pathways during HSAD of untreated and pretreated CM.

During AD process, the methanogenic pathways are dictated by three major kinds of substrates: (a) acetate (b) H_2_ and CO_2_ (also sometime formate, secondary alcohol or ethanol) (c) methanol and methylamines or sometime methylsulphides. Acetoclastic methanogenic pathway was observed predominant in RCM and PCM digesters at low TS content as shown in Fig. 5. In RCM digesters at TS 5%, the *Methanosarcina* and *Methanosaeta* were the two acetoclastic methanogens with a proportion of 55.5% and 9.2% respectively signifying that low TS pressure don’t create any potential inhibitor in the digesters. In the PCM digester at 5% TS, the relative abundance of *Methanosaeta* was 3.6%, while *Methanosarcina* was 61.2% showing a remarkable increase in *Methanosarcina* proportion after freezing and thawing pretreatment. During HSAD between TS 10% to TS 20%, a significant change in the methanogenic community was observed over time, due to compaction and high ammonia/VFA concentration in the digesters. The acetate can only be utilized by syntrophic acetate-oxidizing bacteria (SAOB) when the acetoclastic methanogens are absent. However, the SAOB were unable to perform their function because of a higher Gibbs free energy change (△G0′) value (+ 104.5 kJ/mol) resulting a higher acetate level in the system resulting in the blocking of the acetoclastic methanogenic pathway.

The archaeal composition of RCM and PCM digesters changed significantly upon increasing TS content 10% (*Methanosarcina* 44.0%, *Methanosaeta* 12.2%, *Methanobacterium* 7.4%, *Methanobrevibacter* 12.1% and *Methanoculleus* 5.2%) while in PCM digester (*Methanosarcina* 35.9%, *Methanoculleus* 12.9%, *Methanomassiliicoccus* 12.2%, *Norank_C_Bathyarchaea* 16.7%, *Methanosaeta* 3.0%, *Methanobacterium* 9.9%). The new structure was partially dominated by hydrogenotrophic methanogens. Further increase in TS from 15 to 20% the severe toxicity to acetoclastic methanogens was observed due to high ammonia and VFA concentrations resulting a decreased H_2_ and CO_2_ availability to hydrogenotrophs. At such higher TS concentrations, the population of methylotrophs (especially, *Methanomassiliicoccus)* was observed significantly higher in RCM (18.7% at 15% TS and 17.4% at TS 20%) and PCM (45.5% at TS 15% and 42.1% at TS 20%) digesters because of their highly resistive nature towards the potential inhibitors. These results are in accordance with the previous HSAD studies in sludge^49^, swine manure^43^ and food waste^57^.

Figure 5 depicts an excellent example of changes in methanogenic pathways during the HSAD of freezing and thawing pretreated and untreated CM with increasing solid content. As we went through the archaeal abundance again, we noticed that mainly two species were involved in acetoclastic methanogensis, i.e. *Methanosarcina* and *Methanosaeta* from which *Methanosaeta* is considered a strict acetoclastic archaeon while *Methanosarcina* is a versatile and can utilize CO_2_, H_2_ as well as methylated compounds. A higher abundance of these two genera was responsible for acetoclastic methanogenesis. The hydrogenotrophic methanogens produce biomethane from the reduction of H_2_ and CO_2_. Most of the archaea detected were hydrogenotrophic methanogens. The genera *Methanoculleus*, *Norank_C_Bathyarchaea*, *Methanobacterium*, *Methanospirillum*, *Methanobrevibacter*, *Methanosphaera*, *Methanomicrobia*les, *Candidatus_Methanoplasma*, *Methanofollis* and *Methanolinea* were responsible for hydrogenotrophic pathway. The genera *Methanomassiliicoccus* and *Candidatus Methanomethylophilus* were H_2_ dependent methylotrophs that utilized methylated compounds and were responsible for methylotrophic methanogenesis.

## Conclusion

The freezing and thawing pretreatment of CM not only reduced hardly biodegradable organics significantly but also enhanced hydrolysis rate and biomethane yield. Cellulose contents were reduced by 17.8%, hemicellulose 16.3% and lignin 13.3% during pretreatment. The maximum methane yield of 487 mL g^− 1^VS was obtained at TS 5% in PCM digester followed by TS 10% and TS 15% digesters. Least inhibition was observed in TCM digesters in comparison with RCM. Acetoclastic methanogens were found dominant in RCM digesters and were easily restrained by high ammonia and VFA concentrations. The pathway was shifted to hydrogenotrophic and methylotrophic methanogenesis with increasing solid concentration. Freezing and thawing could prove an energy efficient and eco-friendly pretreatment technique for lingo-cellulosic rich organic wastes in countries having freezing and thawing weather conditions.

## Data Availability

The original contributions presented in the study are included in the article. Further inquiries can be directed to the corresponding authors.
